# Promiscuity in Lichens Follows Clear Rules: Partner Switching in *Cladonia* Is Regulated by Climatic Factors and Soil Chemistry

**DOI:** 10.3389/fmicb.2021.781585

**Published:** 2022-01-31

**Authors:** Zuzana Škvorová, Ivana Černajová, Jana Steinová, Ondřej Peksa, Patricia Moya, Pavel Škaloud

**Affiliations:** ^1^Department of Botany, Faculty of Science, Charles University, Prague, Czechia; ^2^Museum of West Bohemia in Pilsen, Pilsen, Czechia; ^3^Botánica, ICBIBE, Fac. CC. Biológicas, Universitat de València, Valencia, Spain

**Keywords:** lichens, *Cladonia*, *Asterochloris*, photobiont, symbiosis, specificity, green algae

## Abstract

Climatic factors, soil chemistry and geography are considered as major factors affecting lichen distribution and diversity. To determine how these factors limit or support the associations between the symbiotic partners, we revise the lichen symbiosis as a network of relationships here. More than one thousand thalli of terricolous *Cladonia* lichens were collected at sites with a wide range of soil chemical properties from seven biogeographical regions of Europe. A total of 18 OTUs of the algal genus *Asterochloris* and 181 OTUs of *Cladonia* mycobiont were identified. We displayed all realized pairwise mycobiont–photobiont relationships and performed modularity analysis. It revealed four virtually separated modules of cooperating OTUs. The modules differed in mean annual temperature, isothermality, precipitation, evapotranspiration, soil pH, nitrogen, and carbon contents. Photobiont switching was strictly limited to algae from one module, i.e., algae of similar ecological preferences, and only few mycobionts were able to cooperate with photobionts from different modules. Thus, *Cladonia* mycobionts generally cannot widen their ecological niches through photobiont switching. The modules also differed in the functional traits of the mycobionts, e.g., sexual reproduction rate, presence of soredia, and thallus type. These traits may represent adaptations to the environmental conditions that drive the differentiation of the modules. In conclusion, the promiscuity in *Cladonia* mycobionts is strictly limited by climatic factors and soil chemistry.

## Introduction

At present, we regard lichens as complex ecosystems (Hawksworth and Grube, 2020) consisting of a mostly ascomycete fungal partner, one or more photobionts (algal and/or cyanobacterial), bacterial communities (Uphof, 1925; Grube et al., 2009; Bates et al., 2011), and potentially also basidiomycete yeasts (localized primarily in the thallus cortex) (Spribille et al., 2016) and lichenicolous fungi and other lichen-inhabiting organisms (Arnold et al., 2009). Nevertheless, the main role in dispersion, colonization abilities, and environmental preferences is played by the first two above-mentioned partners: the fungal mycobiont and its main associated photobiont. Such symbiosis is not strictly specific, and the coevolution between the involved partners is limited (Rikkinen, 2003; Lücking et al., 2009; Thüs et al., 2011). Similar evolutionary independent symbiotic associations between phototrophic and heterotrophic organisms are not uncommon in nature. Except for some intracellular interactions (Nowack et al., 2016) or the green hydra (*Hydra viridissima*) symbiosis (Kawaida et al., 2013), most symbiotic organisms show low specificity in some life phases or in extreme conditions (Beck, 2002; Osyczka et al., 2021; Rola et al., 2021), forming a symbiotic relationship with a variety of partners. There are plenty of well-known examples of these interactions: for instance, the corals with zooxanthellae (Baker, 2003; Kemp et al., 2014; Hume et al., 2015), relationships between mycorrhizal fungi and vascular plants (Lapeyrie and Chilvers, 1985; Egerton-Warburton and Allen, 2001; Vandenkoornhuyse et al., 2002), or root nodule bacteria and leguminous plants (Vargas and Graham, 1989; Perret et al., 2000). However, it is worth noting that the symbiotic interactions are tremendously widespread in nature, and we can find examples of specificity levels at both ends of the spectrum.

The relatively low degree of specificity of lichens may be related to their sexual reproduction, during which the mycobiont is dispersed without the photobiont. This dispersal strategy requires an almost immediate contact of the fungal spore with a suitable photobiont (Honegger, 1996) from the environment (Etges and Ott, 2001; Dal Grande et al., 2014), or from the thallus of other lichens (Friedl, 1987; Rikkinen, 2003; Dal Grande et al., 2012). Reproduction provided by asexual propagules has the advantage of simultaneous dispersal of both photobiont and mycobiont partners. However, even if the partners co-disperse, their relationship is not obligatory. Indeed, the exchange of the algal partner has been reported in several studies, proposing ecological adaptation to new habitat conditions as a key driver of a low degree of specificity (Nelsen and Gargas, 2008; Wornik and Grube, 2010).

In many lichens, a mycobiont cooperates with several algal or cyanobacterial lineages, though in most cases, photobiont lineages are closely related, belonging to a single genus (DePriest, 2004). However, a number of host partners, primarily belonging to the family Verrucariaceae (for example, *Hydropunctaria rheitrophila* and other amphibious or endolithic species), are known for their ability to form interactions with many unrelated photosynthetic partners (Thüs and Schultz, 2008; Thüs et al., 2011). Another example may be the parasitic *Diploschistes muscorum*, which can involve photobionts belonging to three different genera (Friedl, 1987; Wedin et al., 2016; Osyczka et al., 2021). Since a majority of mycobionts exhibit rather specific relationships with their algal partners, it is possible that promiscuity of partner choice negatively affects otherwise well-tuned partner communication, metabolite exchange (Ten Veldhuis et al., 2020), and the reciprocal coordination of growth and reproduction. On the other hand, high specificity between a single mycobiont and photobiont partner could be disadvantageous in some cases. For example, there is a much lower probability of finding a compatible partner in the case of sexual reproduction. In addition, high specificity should narrow the ecological amplitude of the symbiotic association, limiting its distribution and ability to grow in diverse habitats. In contrast, a mycobiont cooperating with several algal partners may occur across a wider range of conditions (Blaha et al., 2006). Such an exchange of partners is usually connected with different environmental conditions in which the lichen is present (Fernández-Mendoza et al., 2011; Peksa and Škaloud, 2011). Accordingly, the distribution of lichen symbiotic association is then restricted by the intrinsic limits of the mycobiont. We can observe this relationship also from the opposite point of view – the photobiont cooperating with several fungal partners may expand the spectrum of conditions in which it can survive.

The principal aim of this study is to uncover the limits of symbiotic associations on both climatic and habitat scales and to identify the trade-offs between high specificity and promiscuity. More specifically, we ask questions such as: How promiscuous are the symbiotic partners in general? Do the symbiotic partners broaden their distribution range along environmental gradients by loosening their specificity? Which factors impact the boundaries of partner cooperation? Is the choice of photobionts influenced by mycobiont functional traits? Understanding the factors that affect the symbiotic relationships between photobionts and mycobionts should help us to elucidate the mechanisms underlying the ecological uniqueness of lichens growing in extreme environmental conditions where they predominate over the vascular plants.

To answer the above mentioned questions, we used a genus *Cladonia* as a model and analyzed the symbiotic relationships in *Cladonia* terricolous communities growing in a broad spectrum of both climatic and habitat conditions. *Cladonia* represents a sub-cosmopolitan genus (Ahti, 2000; Ahti et al., 2013) with approximately 500 described species (Stenroos et al., 2002; Pino-Bodas and Stenroos, 2020) differing by a number of functional traits, including the frequency of sexual and asexual reproduction, production of secondary chemical metabolites (Stenroos et al., 2002) and thallus type. It forms the partnership with the green algal genus *Asterochloris* (Pino-Bodas and Stenroos, 2020) including 18 species described so far (Škaloud and Peksa, 2010; Škaloud et al., 2015) and several yet undescribed species-level lineages, pointing to the fact the currently described diversity of this genus is clearly underestimated (Pino-Bodas and Stenroos, 2020; Kosecka et al., 2021).

## Materials and Methods

### Sampling

A total of 1,120 lichen thalli were collected within the seven different biogeographic regions of Europe (Mediterranean, Temperate, Atlantic, Boreal, Arcto-Alpine, Pannonian, and Black Sea). Regions were based on the official map of European Biogeographical Regions provided by the European Environment Agency.

In each region, a total of 8 study plots were established, each with an area of 100 m^2^. The plots were not closer than 5 km to each other. Overall, the dataset encompassed 56 sites spanning a latitudinal gradient from 39°45′ to 69°37′ N and covering an east-west distance from 5°3′ W to 32°12′ E, respectively. The plots were selected to present non-forest sites with well-developed *Cladonia* lichen communities, including at least three different morphospecies. In addition, we aimed to span a wide range of substrate pH in each region, selecting four alkaline (e.g., limestone, basalt, diabase, and calcareous soils) and four acidic (e.g., granite, schist, and siliceous sands) plots according to European Geological Data Infrastructure^1^. A total of 20 lichen thalli were collected from each plot, covering all detected morphospecies in equal proportions. The *Cladonia* specimens were first determined in the field and later in the laboratory using standard microscopic and chemical methods, including thin-layer chromatography (TLC), according to Orange et al. (2001).

### Environmental Data

Soil chemistry and climatic data were obtained for every study plot. Nine soil samples per plot were collected, pooled, and frozen at −20°C. pH values and the contents of the following elements and compounds were measured: total nitrogen, total carbon, NH_4_^+^, N-NH_4_^+^, NO_3_^–^, N-NO_3_^–^, NO_2_^–^, N-NO_2_^–^, and P-PO_4_^–^. The ratios between C, N, and P are closely related with the soil’s ecological structure, processes, and functions and are indicators of nutrient dynamic in soil (Zedler, 2000; Zhang Z. S. et al., 2013). The samples were examined by the Czech Geological Survey. To measure pH, 10 g of homogenized soil were soaked in 100 ml of distilled water for 24 h, after which pH was measured using a Cyberscan pH11 pH meter (Eutech Instruments, United States) with a standard glass electrode. For the elemental carbon and nitrogen analysis, the samples were ground, homogenized and injected into the combustion tube of the Flash 2000 analyzer by an automatic dispenser. There, the sample was burned with a stream of pure oxygen at 1,000°C. The resulting nitrogen and carbon oxides were fed to a moisture removal separation column and the oxide content was determined with a conductivity detector. Eager Xperience software (Thermo Fisher Scientific) was used to evaluate the signal (Nelson and Sommers, 1996). For ion determination, samples were homogenized, sieved and dried at 70°C. To prepare the extract, 5 g of soil was weighed and extracted in a rotary shaker with 50 ml of 0.5 M potassium sulfate solution for 30 min. The samples were then filtered and the clear solution was analyzed by flow injection analysis with spectrophotometric detection on a QuikChem FIA+ 8000 Series instrument. The results were evaluated with Omnion 3.0 software (Ammerman, 2001; Egan, 2001). Climatic data were obtained from the CHELSA Bioclim database (Karger et al., 2017) at a resolution of 30 arc s (∼1 km). At each sampling site, 19 bioclimatic variables were obtained by applying a 2 km buffer to limit the effects of spatial bias. In addition, we retrieved annual evapo-transpiration values for each sampling site using the Global Potential Evapo-Transpiration (Global-PET) dataset provided at a resolution of 30 arc s (Trabucco and Zomer, 2019). The values were buffered in a similar way to the CHELSA variables.

### DNA Extraction, PCR, and Sequencing

Fresh lichen material was used for DNA extraction following the CTAB protocol (Cubero et al., 1999). Both algal and fungal nuclear internal transcribed spacers (ITS rDNA) were PCR amplified. The algal ITS rRNA gene was amplified using the algal-specific amplification primer nr-SSU-1780 (5′-CTG CGG AAG GAT CAT TGA TTC-3′) (Piercey-Normore and DePriest, 2001) and the universal primer ITS4 (5′-TCC TCC GCT TAT TGA TAT GC-3′) (White et al., 1990). The fungal ITS region was amplified using the fungal-specific amplification primer ITS1-F (5′-CTT GGT CAT TTA GAG GAA GTA A-3′) (Gardes and Bruns, 1993) and the universal primer ITS4. The newly uncovered algal lineages were additionally characterized by amplification of the actin type I gene (including one complete exon and two introns located at codon positions 206 and 248) (Weber and Kabsch, 1994) using the algal-specific primers ActinF2 Astero (5′-AGC GCG GGT ACA GCT TCA C-3′) and ActinR2 Astero (5′-CAG CAC TTC AGG GCA GCG GAA-3′) (Škaloud and Peksa, 2010).

PCRs were performed in a volume of 20 μl (14.3 μl sterile Milli-Q Water, 4 μl My Taq PCR buffer (Sigma), 0.3 μl of each primer (25 pm/ml), 0.1 μl My Taq DNA Polymerase (Sigma), 1 μl of DNA, not quantified), the PCR conditions were as follows: an initial denaturation at 94°C for 1 min followed by 35 cycles of denaturing at 94°C for 45 s, annealing at 60°C for 1 min and elongation at 72°C for 2 min, with a final extension step at 72°C for 10 min for algal ITS rDNA region, an initial denaturation at 94°C for 1 min followed by 30 cycles of denaturing at 94°C for 45 s, annealing at 54°C for 1 min and elongation at 72°C for 2 min, with a final extension step at 72°C for 10 min for fungal ITS rDNA region and an initial denaturation at 95°C for 5 min followed by 35 cycles of denaturing at 95°C for 1 min, annealing at 61°C for 1 min, and elongation at 72°C for 1 min, with a final extension step at 72°C for 7 min for actin type I region.

The PCR products were quantified on a 1% agarose gel stained with ethidium bromide and purified using Agencourt AMPure XP Magnetic Beads (Beckman Coulter) according to the manufacturer’s protocols. The purified PCR products were sequenced using the same primers at Macrogen in Amsterdam, Netherlands.

### Sequence Alignment and Phylogenetic Analyses

Sequencing reads were assembled and edited using SeqAssem program (SequentiX Software) (Hepperle, 2004). Two different alignments were prepared for the phylogenetic analyses: (i) the fungal ITS rRNA gene alignment and (ii) the algal concatenated ITS rRNA gene + actin gene alignment. Sequences were aligned using MAFFT v.7 software (Katoh and Standley, 2013) under the QINS-I strategy and then manually adjusted in MEGA v.6.0 (Tamura et al., 2013). Seventy-five previously published sequences were added to the algal alignment. These sequences were selected to cover all major algal lineages (Škaloud and Peksa, 2010). After removing identical sequences to speed up the analysis, phylogenetic trees were constructed.

### DNA-Based Species Delimitation

Three different approaches were used for delimiting OTUs based on sequence data: GMYC analysis, a Bayesian implementation of PTP approach (bPTP) (Zhang J. et al., 2013) and Automatic Barcode Gap Discovery method (ABGD) (Puillandre et al., 2012). All analyses were performed on both algal and fungal datasets. First, the ultrametric tree was produced using the BEAST software v.1.10.4 (Suchard et al., 2018) under the assumption of uncorrelated lognormal relaxed molecular clock. The analyses were performed on partitioned datasets using the different substitution models described above and under the constant population size coalescent as the tree prior. Ucld.mean prior was set to exponential distribution with mean 10 and initial value 1. Five MCMC analyses were run for 10 million generations, sampling every 10,000 generations. The outputs were diagnosed for convergence using TRACER v.1.7.1 (Rambaut et al., 2018). Consensus trees were generated using TreeAnnotator v.1.10.4 (Drummond et al., 2012). GMYC analyses were performed on consensus trees under the single-threshold model, using the SPLITS package (Ezard et al., 2009) in R v.4.0.5 (R Core Team, 2020). The bPTP analyses were conducted on the bPTP web Server^2^ based on the ultrametric tree obtained by BEAST. Bayesian species delimitation solutions were produced by this analysis, including the support values.

Finally, we ran the ABGD analysis based on finding the gap in the distribution of pairwise differences among the sequences in the alignment provided. The analysis was performed on the ABDG web site^3^. The range was set of prior intraspecific divergence (P_min_ = 0.001, P_max_ = 0.01), the number of steps (Steps = 10) and Nb bins = 20. We chose Jukes and Cantor, 1969 (JC69) model of DNA evolution (Jukes and Cantor, 1969) and performed four analyses with different values of relative gap width (X set to 0.1, 0.5, 1.0, and 1.5). The final OTU delimitation was selected as a consensus between all three delimitation approaches described above.

### Network Visualization and Modularity Analyses

From the entire dataset of 1,120 samples, 58 samples were excluded due to the lack of photobiont or mycobiont sequences, resulting in a final dataset of 1,062 photobiont-mycobiont symbiotic relations. To explore these relationships in detail, we used the GEPHI software (Bastian et al., 2009) to visualize the network between OTUs delimited as described above. The default algorithms were used and the analysis was performed under the following Force Atlas settings: inertia = 0.1, repulsion strength = 4,000, attraction strength = 5, and maximum displacement = 1. We applied the auto stabilize function with strength = 100, sensibility = 0.2, and gravity = 50. To improve readability and esthetics, a no-overlap algorithm to spread nodes apart was used. The weighted average degree was calculated, and this criterion was applied to adjust the nodes by size. Finally, the GEPHI software was used to perform the modularity analysis using the Louvain method to identify the photobiont-mycobiont modules. The modularity value was calculated on 0.548, and the modules were visualized by colors. Thus, modules of OTUs strongly interacting with each other were visualized. Two nodes (photobiont of the Clade StA3 and associated mycobiont OTU number 148) forming the separate cluster were excluded from the further analysis. In following analyses two datasets were applied. First dataset consisted of both mycobiont and photobiont modules, thus two records of each sample were used (2,122 records). Second dataset contained only samples in which both symbionts belonged to the same module (929 records). Due to high similarity of both results, we present only the results of the second dataset analysis.

Prior to module assignment, we performed isolation by distance (IBD) analyses based on fungal phylogenetic, algal phylogenetic, and geographic distances. Phylogenetic distances were computed from phylogenetic trees (as described above). IBD analyses were performed by Mantel tests in R, using the package vegan (Dixon, 2003). Both analyses identified significant relationships, but with a very weak strength (r Mantel statistic 0.07 and 0.16, respectively).

### Variation Partitioning

Two variation partitioning analyses were performed. First, we evaluated the relative effects of geography, climate, substrate chemistry, and the symbiotic partner on the variance in photobiont as well as mycobiont diversity (Borcard et al., 1992). Second, we tested whether the modules identified by the modularity analyses (see above) can be differentiated by geography, climate, and substrate chemistry. The entire procedure was done in R v. 4.0.5 (R Core Team, 2020) using base functions and the packages vegan (Dixon, 2003), SoDA (Chambers, 2013), phytools (Revell, 2012), geiger (Pennell et al., 2014), ape (Paradis and Schliep, 2019), ade4 (Dray and Dufour, 2007), and geosphere (Hijmans, 2019). When using the photobiont/mycobiont diversity as response variables, the phylogenetic distances were computed from phylogenetic trees (as described above) and transformed into principal coordinates analysis (PCoA) axes. Concerning the explanatory variables, geographical distance values (latitude and longitude) were transformed to the principal coordinates of neighbor matrices (PCNM) vectors representing the geographical distances at various spatial scales (Borcard et al., 2004). Climate was characterized by 19 bioclimatic variables plus annual evapo-transpiration as specified above. Substrate chemistry was characterized by pH and contents of nine elements and compounds as specified above. Explanatory variables included in the variation partitioning analyses were selected either by the forward selection of redundancy analysis (RDA) or by transforming the variables into principal component analysis (PCA) axes, followed by the selection of most important axes by the broken-stick distribution (Jackson, 1993) using the bstick function.

Climatic and substrate chemistry variables selected by the forward selection as the best explanatory variables to differentiate among the modules were visualized by boxplots.

Bonferroni-corrected (*p* < 0.05) Wilcoxon signed rank test (Wilcoxon, 1992) was used to test the significance of selected variables to differentiate among the modules, generated all pairwise comparisons, computed *p*-values from Wilcoxon test for all comparisons and tested all modules, pairwise. Then, we tested which groups are significantly different.

Finally, we performed PCA analyses of localities based on their climatic and soil chemical data to show the differences among the modules. We analyzed the same explanatory variables selected by the forward selection and visualized by boxplots (see above).

### Functional Traits of Mycobionts

A set of anatomical, morphological, and chemical traits were recorded for each lichen morphotype investigated (Supplementary Table 2). These are as follows: the presence of sexual reproduction structures (common/rare), the thallus type (dimorphic/reindeer/reindeer like/squamulose), the presence of vegetative reproduction propagules (soredia, granules, and squamules), the character of podetia (corticate/ecorticate), main chemical compound in the cortex (atranorin/usnic acid/none of them), and the color of apothecia (brown/red) (James, 2009; Ahti et al., 2013). In case of assignment of the sample to the known morphospecies the data were taken from the literature sources, in case of unclear morphotype the observations were made separately for each sample.

The significance of each trait to differentiate among the modules was tested in R by Chi-square test, using packages fastDummies (Kaplan, 2020), fmsb (Nakazawa, 2012), and corrplot (Wei et al., 2017).

## Results

### Diversity of Mycobionts and Photobionts

**FIGURE 1:**
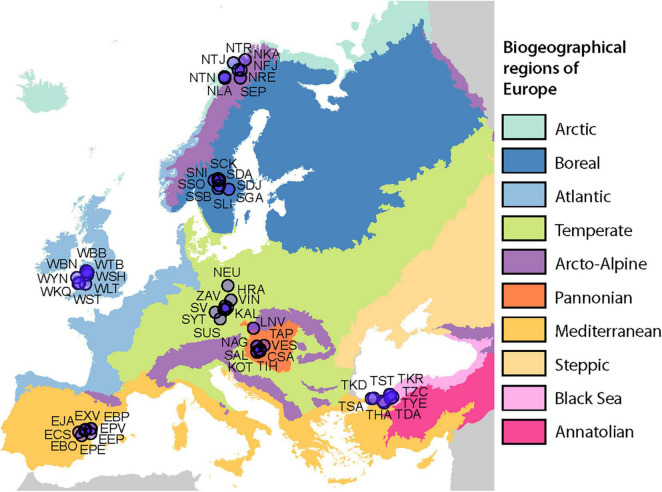
Sampling design. Eight sites were sampled in seven of the biogeographical regions of Europe. Map copyright holder: Council of Europe (CoE), Directorate-General for Environment (DG ENV).

On 56 sampling sites (Figure 1 and Supplementary Table 1), a total of 1,120 samples were collected. We detected 65 *Cladonia* morphotypes including subspecies and varieties (Supplementary Table 2).

From 499 fungal genotypes a total of 181 *Cladonia* OTUs were identified based on species delimitation methods. OTUs were found 1–45× (Supplementary Figure 1). These OTUs were assigned to their respective clades and subclades following division and nomenclature suggested by Stenroos et al. (2019).

From 76 genotypes a total of 18 *Asterochloris* OTUs were identified based on species delimitation methods (Supplementary Figure 2). The *Asterochloris* genotypes belonged to the previously known species *Asterochloris irregularis*, *Asterochloris glomerata, Asterochloris italiana, Asterochloris lobophora, Asterochloris mediterranea, Asterochloris woessiae, Asterochloris magna, Asterochloris erici*, and *Asterochloris antarctica* (Škaloud and Peksa, 2010; Peksa and Škaloud, 2011; Moya et al., 2015; Škaloud et al., 2015), seven clades named according to Kosecka et al. (2021) and two new lineages we labeled *A.* aff. *italiana* and clade A15.

### Variation Analyses

**FIGURE 2:**
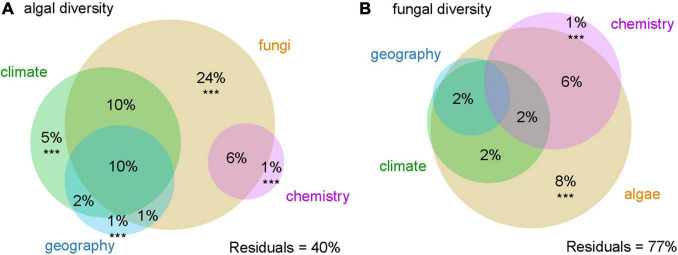
Variation partitioning of **(A)** algal and **(B)** fungal diversity. Variables significantly contributing to the variation explained (*p* < 0.001) are marked with ***.

The majority of variation in algal and fungal diversity was explained by algal-fungal relationship (24% of algal diversity was explained by the associated fungus and 51% in combination with other variables, 8% of fungal diversity by the associated alga, and 20% in combination with other variables, respectively). The algal variation was also much better explained by the climate (5% independent effect and 27% in combination with other variables) than the fungal one (6% in combination with other variables). Soil chemistry accounted for only a small proportion of the variation (1% independently, 7% in combination with other variables in explanation of both algal and fungal diversity) as well as geographical values (14% in combination with other variables in case of algal diversity and only 2% in combination with other variables in fungal diversity) (Figure 2).

### Modularity Analysis

**FIGURE 3:**
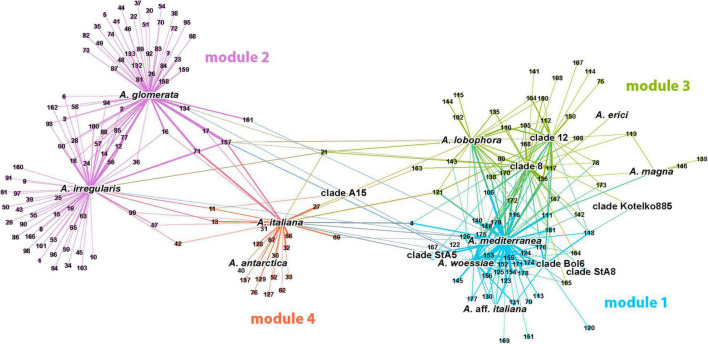
Visualization of the photobiont-mycobiont (marked with photobionts species names and mycobionts OTU numbers, respectively) network resulting from the modularity analysis. No-overlap algorithm was used to spread nodes apart. Four major modules were found and they are distinguished by color.

Four major modules (= clusters) were defined by the cluster analysis (Figure 3). In the following text we refer them to as module 1 (*A. mediterranea*), module 2 (*A. glomerata*), module 3 (*A. lobophora*), and module 4 (*A. italiana*). In the first module (displayed in blue color), the 34 fungal OTUs were associated with algal strains *A. mediterranea*, *A. woessiae*, *A.* aff. *italiana*, clade A15, clade Bol6, and StA8 (19.5% of nodes). Mycobionts were the members of clades Crustaceae and Cladonia (all recorded subclades except subclade Macropus).

The second module (in purple color) represented the largest cluster (47.7% of nodes), and was formed by the photobiont OTUs coinciding with lineages *A. glomerata* and *A. irregularis* and 91 associated mycobiont OTUs. The predominant part of them was found in the symbiosis with only one of this algal OTU. Mycobionts belonged to the clades Crustaceae, Erythrocarpae, Perviae, Arbuscula, Impexae, and Cladonia (subclades Macropus, Gracilies, Foliaceae, and Cladonia).

The third module (in green color) was more connected to module 1, but a similar trend was maintained again: the majority of relationships between OTUs were implemented within the clusters. This module was centered around *A. lobophora, A. erici, A. magna*, and clades 8, 12, and Kotelko885 (21.5% of nodes). A total of 36 associated mycobionts belonged to the clades Erythrocarpae and Cladonia (subclades Gracilies, Helopodium, Cladonia, and Ascyphiferae). The fourth module (in orange color) was centered around algal OTUs *A. italiana, A. antarctica*, and clade StA5 (11.3% of nodes). The associated mycobionts (19 OTUs) belonged for the most part to the clades Unciales, Erythrocarpae, Perviae, Impexae, and Cladonia (subclades Gracilies and Firmae).

**FIGURE 4:**
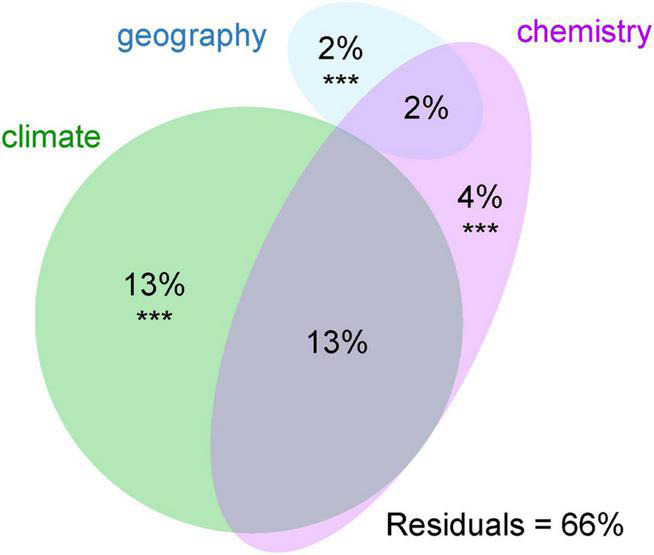
Variation partitioning of the effects of environmental variables on the photobiont-mycobiont modules found by modularity analysis. Variables significantly contributing to the variation explained (*p* < 0.001) are marked with ***.

**FIGURE 5:**
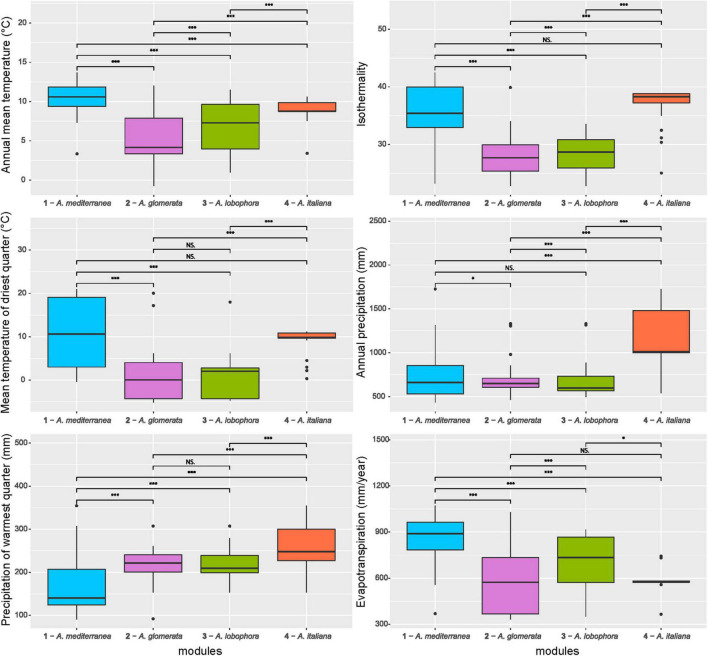
Comparison of climatic variables between the photobiont-mycobiont modules. Significance levels, calculated by Wilcoxon test, are given: “***” – *p* < 0.001, “*” – *p* < 0.05, “NS” – not significant. Bold lines represent the median and whiskers account for the 95% confidence interval of the data. Outliers appear as circles.

**FIGURE 6:**
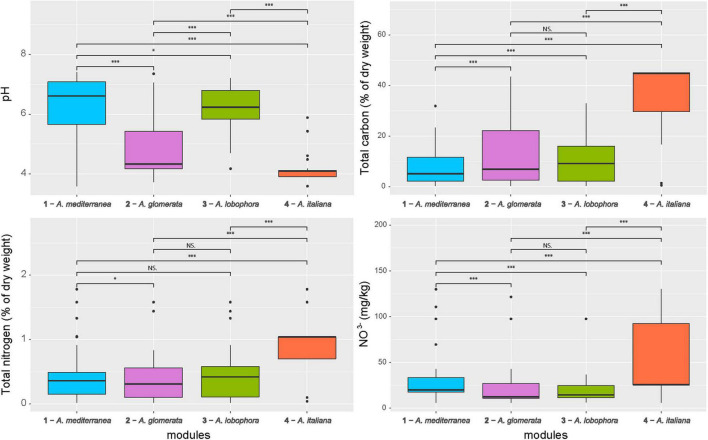
Comparison of soil properties between the photobiont-mycobiont modules. Significance levels, calculated by Wilcoxon test, are given: “***” – *p* < 0.001, “*” – *p* < 0.05, “NS” – not significant. Bold lines represent the median and whiskers account for the 95% confidence interval of the data. Outliers appear as circles.

The majority of variation between modules was explained by climatic data only (13%) and a combination of climatic and chemistry data (12%). Only 2% of the variability was explained by geography (Figure 4). The significant differences between clusters were discovered in the following climatic variables: annual mean temperature, isothermality, mean temperature of driest quarter, annual precipitation, precipitation of warmest quarter, and evapotranspration (Figure 5). The significant differences were also discovered in modules preferences in soil pH, total carbon, total nitrogen, and nitrate contents in the soil (Figure 6).

**FIGURE 7:**
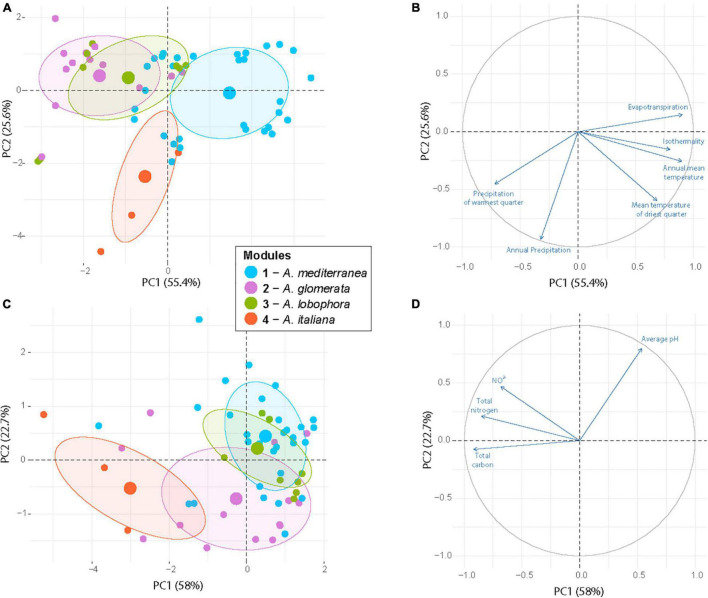
Principal component analysis of the sampling sites based on six climatic variables **(A,B)** and four soil properties **(C,D)** that significantly differ between the photobiont-mycobiont modules. Large circles represented group centroids. The modules are distinguished by color.

Each module can be clearly differentiated by the combination of climatic and soil chemistry preferences (Figure 7). Module 4 (*A. italiana*) differs in its both chemical and climatic demands compared to the others, preferring humid conditions with high levels of nutrients. Modules 2 (*A. glomerata*) and 3 (*A. lobophora*) have similar requirements for climatic conditions, but module 2 prefers distinctly lower pH (Figure 6). Modules 1 (*A. mediterranea*) and 3 have similar chemical demands, but they differ in their climatic preferences. Specifically, module 1 prefers warmer regions with high isothermality (Figure 5). The low number of relationships between the modules points out the importance of the above-mentioned factors in shaping the distribution of lichen associations.

### Functional Traits of Mycobionts

**FIGURE 8:**
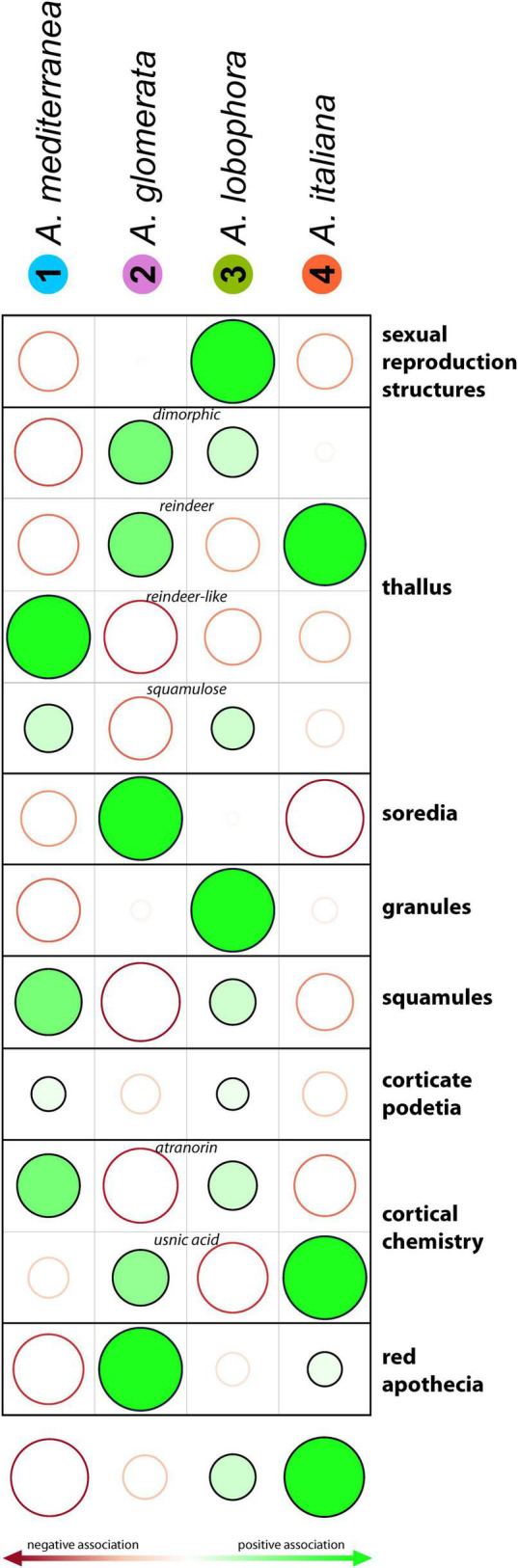
Incidence of lichen morphological traits between the modules. Filled and empty circles display positive and negative associations between the module assignment and the trait values. Stronger associations are shown by larger circles and darker colors. The darker the color, the more distinct the observed value than the expected value if the data were random (calculated by Chi-squared test). The size of the circle is proportional to the amount of the cell contribution.

The significant differences between modules were found in the following morphological, anatomical and chemical traits of lichens: frequency of sexual reproduction, the thallus type, the presence of soredia, granules, and squamules, the presence of cortex on podetia, red color of apothecia and cortical chemistry (Figure 8).

## Discussion

### The Limits of Symbiotic Interactions

How are the symbiotic trade-offs resolved, and what factors play an important role in limiting the algal-fungal promiscuity? In accordance with previous studies (Wirtz et al., 2003; Leavitt et al., 2015, Singh et al., 2017), our results confirm that the lichen association does not represent a strictly specific relationship. However, although this association is relatively flexible, the choice of the partner is limited to some extent. We visualized these limits through the projection of the relationships as a network forming four modules, which are distinguished by their preferences for soil chemistry and bioclimatic conditions. On the other hand, geographical parameters did not play a significant role in module differentiation. Several studies revealed that the identity of a partner and climatic variables are key factors in diversity variation, but in some cases the geographical parameters may also play a role (Fernández-Mendoza et al., 2011; Vančurová et al., 2018; Pino-Bodas and Stenroos, 2020).

Why do climate and soil chemistry, especially the pH, represent key factors distinguishing the modules? The influence of pH on lichens is a traditionally studied phenomenon, and, indeed, the pH has been demonstrated repeatedly as one of the most important factors affecting the species composition of a lichen community, as well as the species distribution (Brodo, 1973). For example, several studies focused on the effect of acid rain (Gilbert, 1986), soil pH (Zraik et al., 2018), rock types (Easton, 1994), and type of bark (Vondrák and Liška, 2010; Rosabal et al., 2013) on lichen diversity and ecology. pH affects the compounds and ions present in the environment, such as heavy metals, which may lead to lichen death by their toxic effects (Goyal and Seaward, 1981; Purvis and Halls, 1996). pH also influences the activity of lichen substances, for instance, usnic acid (Cocchietto et al., 2002; Hauck and Jürgens, 2008) and atranorin (Gardner and Mueller, 1981). Furthermore, it has been hypothesized that the algal partner and its photosynthetic apparatus is very sensitive to pH changes (Lechowicz, 1982; Scott and Hutchinson, 1987) and, consequently, the growth of the holobiont is restricted to a narrow pH range. Indeed, our results show that the fungi can pair with alga having similar ecological demands. Algae have a narrow ecological range, for example, they do not show tolerance for a wide range of soil pH values. This ability to alternate the partners within a suitable pool allows the species to reach its own limits. This finding is consistent with the well-known fact that only a minor fraction of lichen species that can grow on substrates with distinctly different pH. Accordingly, the substrate is traditionally used as a key determination character to distinguish some lichen taxa (Resl et al., 2018).

The lichens usually grow at the surface of various substrates to keep the photobiont adequately illuminated. They are thus exposed to various climatic stress factors, and as poikilohydric organisms they are extremely dependent on ambient climatic conditions. Lichens are adapted to continuous alternating wet and dry cycles. During the dry period, their metabolic activity is slowed down. On the other hand, continuous wetting leads to lichen death (Honegger, 2006). The mycobionts do not protect their photobionts from desiccation, but both bionts have to be individually adapted for the wetting-drying cycles (Honegger, 1991; Gasulla et al., 2009). The cell division of the photobiont is usually under the strict control of the mycobiont (Hill, 1994; Del et al., 1998), but during the high humidity period, the photobiont could increase its reproduction speed and escape from the thallus (Slocum et al., 1980). Therefore, the mutual cooperation of both symbionts must be optimized for current climatic conditions. For these reasons, the bioclimatic variables that characterize the temperature and water conditions at the locality (annual mean temperature, isothermality, mean temperature of driest quarter, annual precipitation, and precipitation of driest quarter) were shown to represent important factors significantly dividing the modules. Similar to pH, we did not observe any photobiont and mycobiont OTU distributed over a wide range of climatic conditions.

### Interactions Among the Modules

The modules represented distinct sub-networks of fungal-algal relationships. Although in most cases, mycobiont–photobiont associations were maintained within a single cluster, in several cases, a mycobiont was found capable of interacting with photobionts from different modules, and four fungal OTUs were found to be associated with algae belonging to three different modules: OTU 4 (a reindeer lichen from the clade Crustaceae, present in Temperate and Atlantic regions), OTU 21 (a dimorphic lichen from the clade Erythrocarpae, present in Temperate, Arcto-Alpine, and Boreal regions), OTU 121 (a dimorphic lichen from the clade Cladonia, subclade Foliaceae, present in Temperate, Atlantic, and Black Sea regions), and OTU 157 (a dimorphic lichen from the clade Cladonia, subclade Cladonia, present in all regions except Mediterranean).

In the case of OTUs 157 and 121, we observed that at each locality, these two fungal genotypes cooperated with photobiont belonging to only a single module. The reason for this may be that the selected photobiont is the most preferred partner at the locality, the one best adapted to the given local conditions. Alternatively, the preferred photobiont might be absent at the locality, and thus the mycobiont is forced to settle for a suboptimal partner. We could hypothesize that the mycobiont OTU 121 prefers the symbiosis with algae from the module 3 (*A. lobophora*), but at localities where no algae from that module are present, it can establish a relationship with algae belonging to other modules (i.e., the modules 1 – *A. mediterranea* and 4 – *A. italiana*). However, our assumptions are based only on the findings of this single research study. To get a complete picture it would be necessary to verify the presence of lichen algae in the soil of the locality. In any case, these OTUs represent remarkable mycobionts with a broad ecological amplitude, which can cope with different climatic and environmental conditions and widen their niches through photobiont switching.

Several studies published so far have pointed out the problematic species delimitation using the ITS rDNA marker, which is generally used for fungal species delimitation (Fontaine et al., 2010; Kotelko and Piercey-Normore, 2010; Pino-Bodas et al., 2013). Pino-Bodas et al. (2013) mentioned the necessity of combining it with other molecular loci. Due to the nature of this study, the ITS rDNA was used to distinguish OTUs, keeping in mind that these do not represent well-defined species (Ahti, 2000). However, in the aforementioned cases of OTUs cooperating with three modules, the study of Stenroos et al. (2019) shows that their ITS rDNA sequences form a single, well-defined species based on multigene analysis. Therefore, we can exclude the possibility of an incongruent phylogenetic signal among the genes.

### Module Differentiation by Functional Traits

Along with climatic and soil chemistry preferences, the modules also differed in morphological and functional traits of lichens. Module 3 (*A. lobophora*) showed a significantly higher rate of sexual reproduction compared to the expected value if the data were random, whereas in modules 1 (*A. mediterranea*) and 4 (*A. italiana*), the frequency was lower. This phenomenon could reflect the availability of photobionts in the environment. If the cooperating alga is rarely present in the soil, asexually reproducing lichens, dispersing both partners simultaneously, might be favored.

Module 4 that was commonly present at localities with a lower pH, was characterized by a frequent production of usnic acid. Indeed, the activity of this acid is higher just at a low pH (Gardner and Mueller, 1981). Bačkor et al. (1992) demonstrated that the growth of the photobiont is also affected by this acid, i.e., the mycobiont can only be forced to cooperate with algal genotypes that are able to tolerate its activity. Similarly, in module 2 (*A. glomerata*), which also prefers low pH, we found significantly fewer lichens containing atranorin in the cortex, the substance showing a higher activity at higher pH (Gardner and Mueller, 1981).

Pyatt (1973) and Hamlett et al. (2011) described the formation of soredia as an adaptation to successful dispersion in humid conditions. Similarly, Armstrong (1991) reported a positive correlation between the rate of soredia dispersal and precipitation, considering raindrops as a possible transmission route. On the other hand, he described atmospheric humidity as the main factor negatively affecting soredia and considered the cycles of wet and dry periods as optimal conditions for soredia dispersal. During wet periods, soredia accumulate on the lichen surface while they are released in large quantities during dry periods. Accordingly, Tormo et al. (2001) also observed a higher rate of soredia release in the dry periods of the year compared to the wet months. In our case, we detected distinctly fewer soredia-producing species in the module 4 (*A. italiana*), which was characterized by high annual precipitation and low evapotranspiration. Such characteristics indicate a relatively stable and humid climate, which may be disadvantageous for sorediate species.

Steinová et al. (2019) studied four *Cladonia* species to assess if the reproductive and dispersal strategies affect mycobiont–photobiont association patterns. They found the sorediate species associating exclusively with *A. glomerata* and *A. irregularis*, in accordance with the increased presence of soredia in our module 2.

## Conclusion

Our study identified the limits of symbiotic associations in terricolous *Cladonia* lichen communities, identifying climate and soil chemistry as the major factors limiting the distribution of holobionts. Contrary to the commonly held view of lichen symbiosis dynamics, we demonstrated the limited ability of a fungal host to increase its ecological niche through algal symbiont switching. Instead, the frequently reported symbiont switches (Piercey-Normore and DePriest, 2001; Yahr et al., 2006; Rolshausen et al., 2020) may simply result from the absence of a preferred algal partner at a given locality and its replacement by another alga from a compatible ecological module. Such holobiont ecological specialization may also explain the unsuccessful results of lichen transplantation experiments (Higgins et al., 2015; Williams et al., 2017).

## Data Availability Statement

The sequences generated for this study were deposited in GenBank (https://www.ncbi.nlm.nih.gov/genbank/; accession numbers are listed in Supplementary Table 2). The alignments have been deposited in Mendeley Data: https://doi.org/10.17632/zyb8fct4dt.1. Results of chemical analyses are provided in Supplementary Table 1.

## Author Contributions

All authors contributed to conceptualizing the study. IČ, JS, OP, and PM provided the sampling. ZŠ performed the molecular work. PŠ, ZŠ, and JS provided the analyses. OP took care for the chemical analyses of soil samples. PŠ provided the resources, funding, and supervision. All authors contributed to writing, reviewing, and editing.

## Conflict of Interest

The authors declare that the research was conducted in the absence of any commercial or financial relationships that could be construed as a potential conflict of interest.

## Publisher’s Note

All claims expressed in this article are solely those of the authors and do not necessarily represent those of their affiliated organizations, or those of the publisher, the editors and the reviewers. Any product that may be evaluated in this article, or claim that may be made by its manufacturer, is not guaranteed or endorsed by the publisher.

## Footnotes

1
http://www.europe-geology.eu/

2
http://species.h-its.org/ptp/

3
https://bioinfo.mnhn.fr/abi/public/abgd/abgdweb.html

## Supplementary Material

Supplementary Table 1 List of localities included in this study, including the results of soil chemical analyses.

Supplementary Table 2 List of all investigated 1,120 lichen samples, specifying their morphospecies determination, genetic affiliation to fungal and algal OTUs, clustering to the modules, recorded anatomical, morphological and chemical traits, and GenBank accession numbers.

Supplementary Figure 1 DNA species delimitation of fungi. The topology shown corresponds to the Bayesian ultrametric tree obtained in BEAST. The results of three species delimitation analyses (GMYC, bPTP, and ABGD) are shown along the tree, with the final OTU delimitation selected as a consensus between all delimitation approaches. Each color corresponded with the different fungal species delimited. *Cladonia* clades and subclades Stenroos et al. (2019). On the right, the total number of samples per each genotype is indicated by a barplot. The second vertical column represents which module from Figure 3 does each OTU get assigned to (displayed by colors: blue, Module 1; purple, Module 2; green, Module 3; orange, Module 4).

Supplementary Figure 2 DNA species delimitation of algae. The topology shown corresponds to the Bayesian ultrametric tree obtained in BEAST. The results of three species delimitation analyses (GMYC, bPTP, and ABGD) are shown along the tree, with the final OTU delimitation selected as a consensus between all delimitation approaches. Each color corresponded with the different algal species delimited. On the right, the total number of samples per each genotype is indicated by a barplot. The second vertical column represents which module from Figure 3 does each OTU get assigned to (displayed by colors: blue, Module 1; purple, Module 2; green, Module 3; orange, Module 4).
