# Supplementary figures and images for: Promiscuity in Lichens Follows Clear Rules: Partner Switching in Cladonia Is Regulated by Climatic Factors and Soil Chemistry
Source: Front Microbiol. 2022 Jan 31;12:781585. doi: 10.3389/fmicb.2021.781585 (PMC8841807; doi:10.3389/fmicb.2021.781585)

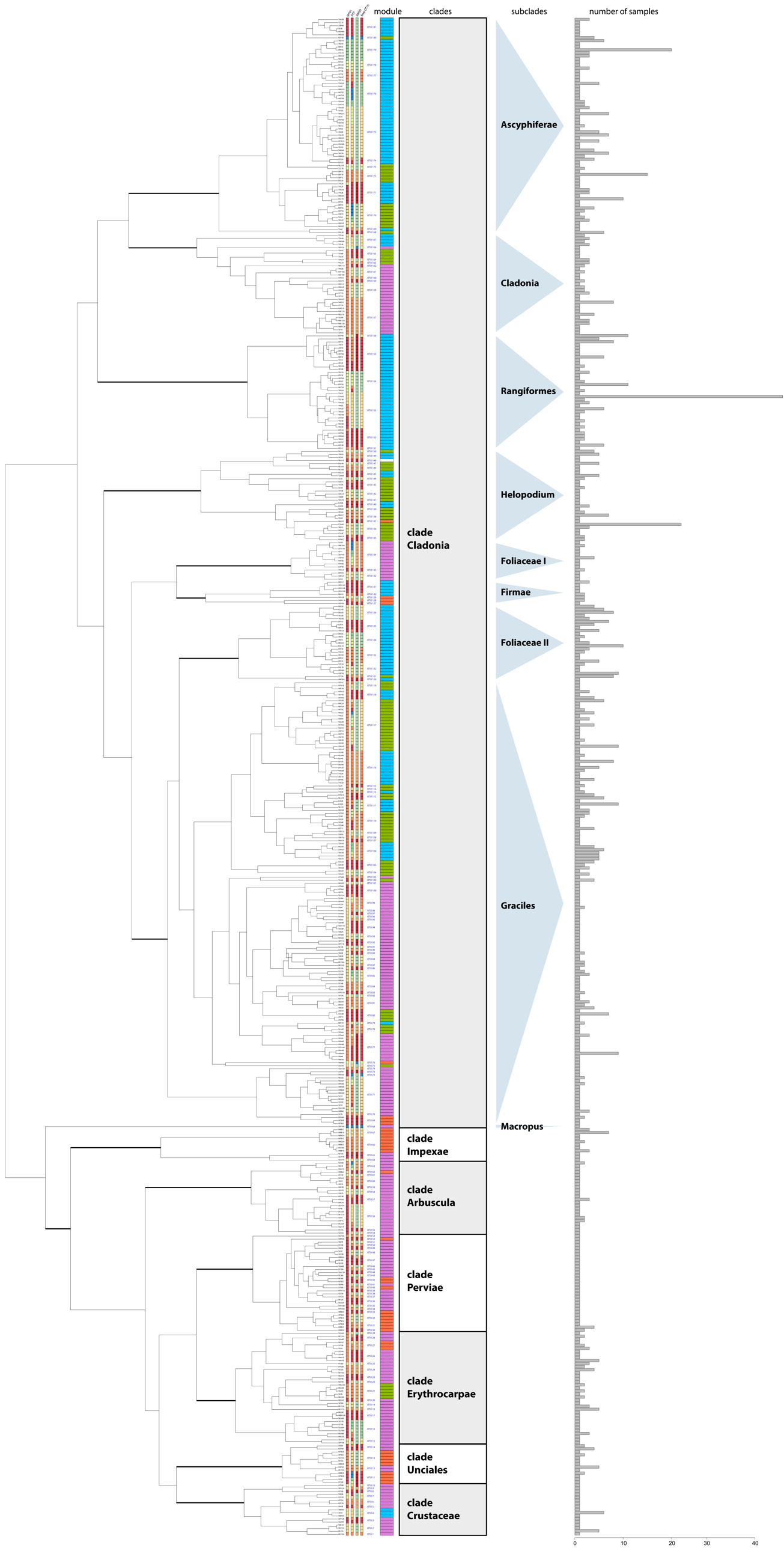

Supplement: Supplementary Figure 1 — DNA species delimitation of fungi. The topology shown corresponds to the Bayesian ultrametric tree obtained in BEAST. The results of three species delimitation analyses (GMYC, bPTP, and ABGD) are shown along the tree, with the final OTU delimitation selected as a consensus between all delimitation approaches. Each color corresponded with the different fungal species delimited. Cladonia clades and subclades Stenroos et al. (2019). On the right, the total number of samples per each genotype is indicated by a barplot. The second vertical column represents which module from Figure 3 does each OTU get assigned to (displayed by colors: blue, Module 1; purple, Module 2; green, Module 3; orange, Module 4). [file Image_1.pdf]

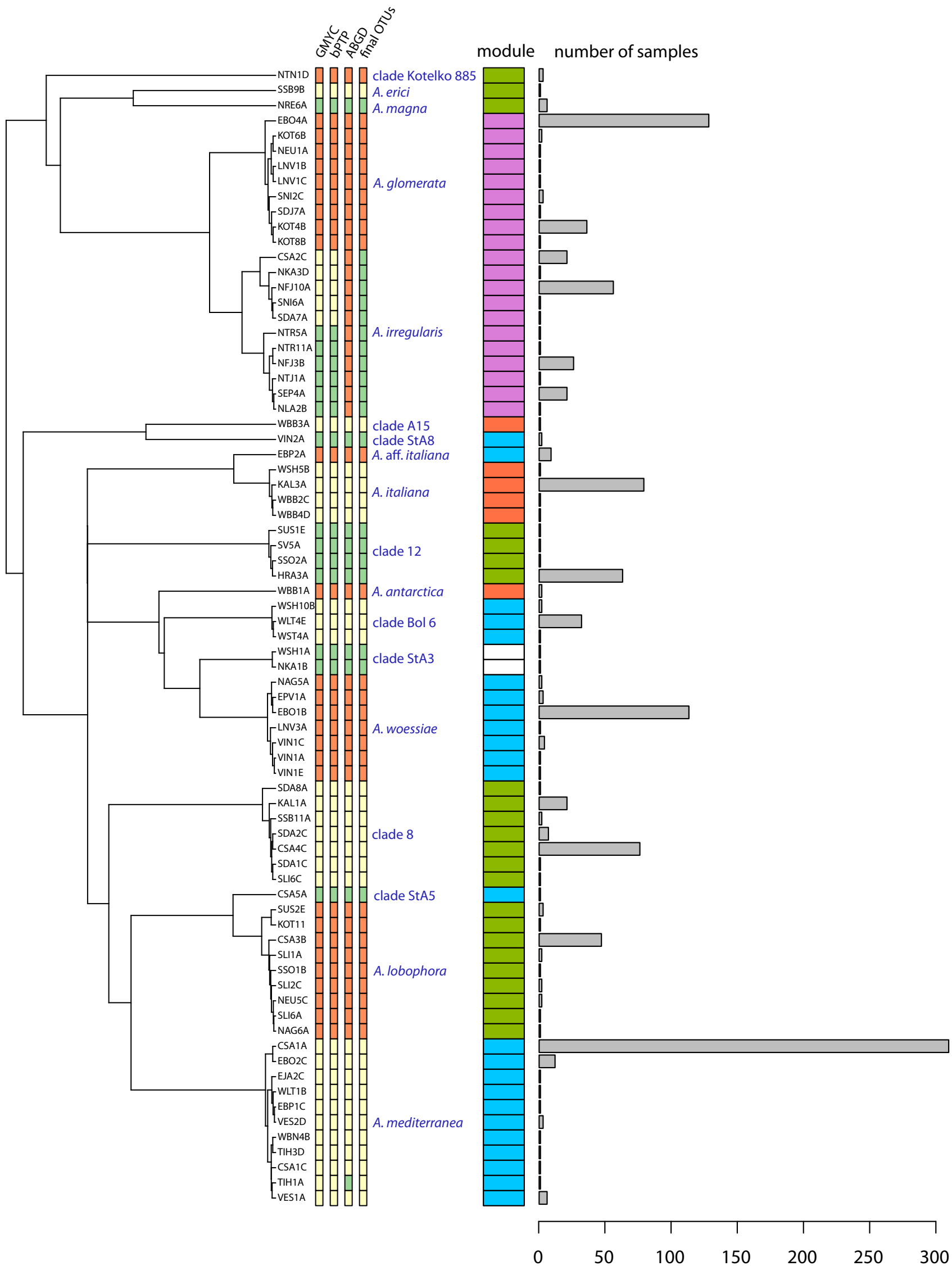

Supplement: Supplementary Figure 2 — DNA species delimitation of algae. The topology shown corresponds to the Bayesian ultrametric tree obtained in BEAST. The results of three species delimitation analyses (GMYC, bPTP, and ABGD) are shown along the tree, with the final OTU delimitation selected as a consensus between all delimitation approaches. Each color corresponded with the different algal species delimited. On the right, the total number of samples per each genotype is indicated by a barplot. The second vertical column represents which module from Figure 3 does each OTU get assigned to (displayed by colors: blue, Module 1; purple, Module 2; green, Module 3; orange, Module 4). [file Image_2.pdf]
